# A qualitative study on health stigma and discrimination in the first year of the COVID-19 pandemic: Lessons learnt from a public health perspective

**DOI:** 10.3389/fpubh.2023.1143640

**Published:** 2023-03-01

**Authors:** Chou Chuen Yu, Bernard Tang, James Alvin Low, Mathews Mathew, Sharon Straus, Christine Fahim

**Affiliations:** ^1^Geriatric Education and Research Institute Ltd., Singapore, Singapore; ^2^Department of Geriatric Medicine, Khoo Teck Puat Hospital, Singapore, Singapore; ^3^Institute of Policy Studies, National University of Singapore, Singapore, Singapore; ^4^Li Ka Shing Knowledge Institute, St. Michael's Hospital, Unity Health Toronto, Toronto, ON, Canada

**Keywords:** COVID-19, stigma, fear, misinformation, discrimination, public health, ageism, healthcare workers

## Abstract

**Background:**

Stigmatisation, misinformation and discrimination have been magnified globally due to the COVID-19 pandemic. The healthcare sector was not spared from this. We conducted a transnational study, using the Health Stigma and Discrimination framework (HSDF) to explore public perception and reactions to the COVID-19 pandemic in a multicultural context. Findings from the Asian arm of the study, sited in Singapore, are reported in this paper.

**Methods:**

This phenomenological research deployed semi-structured informant interviews using non-probability sampling approaches to recruit members of the public. Interviews were coded independently by two researchers and thematic analysis was used to analyse the responses.

**Results:**

Twenty-nine members of the public (23–80 years old) were interviewed between Oct 2020 to Feb 2021. Five major themes were identified: (i) perception of stigma amongst respondents, (ii) experiences of stigma amongst respondents, (iii) views on what drove stigma and misinformation, (iv) facilitators in preventing and reducing stigma and misinformation, and (v) ageist attitudes towards older adults. Overall, construction workers living in dormitories, healthcare workers, and to some extent tourists from China, were perceived to have been stigmatised and shunned by the public. Place-based stigmatisation was common; participants responded by avoiding places that had confirmed cases of COVID-19. Perceived stigma was temporary and not enduring, driven at the outset by fear of being infected. This study also identified the role played by trust in reducing stigmatisation. The relative absence of politicising of issues and high-quality information readily disseminated to the public were reported as factors that could have reduced and prevented stigma and misinformation on the various groups. Ageist attitudes were observed in some participants with older adults being labelled as vulnerable, susceptible to misinformation and being less able to cope during the pandemic.

**Conclusion:**

Through the lens of the HSDF, this study provided an exploratory account of the nature of stigma that resulted from the COVID-19 pandemic in an Asian context. It also shed light on facilitators in preventing and reducing stigma during an outbreak especially the role of trust and communications during a public health crisis.

## 1. Introduction

In January 2020, the WHO declared the COVID-19 pandemic as an international public health emergency (1). To reduce the rate of infection, governments around the world adopted various policies such as the practise of safe distancing and mask wearing, tight control of population mobility (2), nationwide quarantine measures (3) and other emergency preparedness strategies. While providing guidance and direction to manage a rapidly evolving disease, governments also assumed the responsibility of communicating information, risks, and management strategies to the general public and higher risk groups. In the management of public health emergencies, clear, accurate, and transparent communication is critical (4). However, the uncertainties surrounding a novel disease such as COVID-19 have made information sharing challenging (5).

In a highly connected digital era, many people were quickly exposed to misinformation or conflicting information about the virus such as COVID-19 preventative measures, conspiracy theories about the origins of the virus, or misconceptions about one's perceived susceptibility towards the virus (6–8). This is alarming as the perceived accuracy of a single piece of misinformation could increase even with a single exposure (9). Additionally, one's intention to verify information could also be hindered by motivated reasoning in an attempt to protect existing beliefs (10). A multi-country study comparing the impact of exposure to COVID-19 misinformation in the USA, South Korea, and Singapore found that exposure to misinformation had a significant direct association with information avoidance and heuristic processing (7). While cultural and situational differences may affect response towards and interpretation of misinformation, information-seeking behaviour appears to be similar across cultures (7).

Misinformation, also has the potential to instil fear stigmatisation and discrimination against groups such as patients (11) healthcare workers (12, 13), older adults (14) or individuals of Asian descent (14, 15). Health related stigmatisation, or stigmatisation association with health conditions, can have consequences for public health, for instance leading to affected groups avoiding testing, treatment or other health seeking behaviours (16). Additionally, mental health of those stigmatised could also be affected (11, 17–19). Whilst affected groups may not be excluded or rejected out rightly as a result of discrimination, they can still be subjected to stigmatising behaviours that can fall outside the purview of the law such as verbal abuse or gossip (16). As Singapore was one of the initial countries within the Southeast Asian region where COVID-19 spread, many were potentially exposed to misinformation about the origins of COVID-19 due to its novelty (20, 21). Being a multi-racial and multi-religious city-state with various ethnic groups, the rise of misinformation, fear, and stigma in the face of a pandemic poses a threat to social harmony, as previously seen during the 2003 Severe Acute Respiratory Syndrome (SARS) pandemic in 2003 (22). As social cohesion is important in managing a pandemic such as through positive attitudes towards immigrants (23) and other vulnerable groups, understanding how COVID-19 leads to misinformation, fear, and stigmatisation could inform the development of strategies to alleviate these issues in combating future pandemics.

To address this knowledge gap, a qualitative study was conducted to explore the perceptions and reaction of laypersons, to the COVID-19 pandemic in Singapore. This study was part of an international collaborative effort to further explore how misinformation, fear and stigma are contextualised within a cultural, political and global setting in both Canada and Singapore. The design of this study was guided by elements of the Health Stigma and Discrimination Framework (HSDF) (16). The HSDF helps conceptualise the stigmatisation process across a spectrum of socio-ecological determinants. It considers how individual characteristics (such as race, sex, gender, age) overlap and intersect with organisational biases and power structures with communities, organisations, or systems (16). This framework makes it possible to move away from the dichotomous thinking of “us” vs. “them” with regards to stigma and allows for more comprehensive understanding of the construct. More importantly, the HSDF distinguishes between stigmatised experiences and stigma practises. The former leads to an impact of outcomes such as emotional health, social exclusion, reduced access to treatment, while the latter results in fear or misinformation that perpetuates stereotypes and discrimination (16). Findings from this study could help to catalyse the development of appropriate strategies and tools to combat misinformation, fear, and stigma in response to the COVID-19 outbreak.

## 2. Methods

### 2.1. Study design

Colaizzi's phenomenological method (24) was used for this study. In phenomenology, the subjective experiences of participants are understood by returning to the specific life scenes of the participants and exploring their feelings, perceptions, and reactions to the latter. In the context of this study, the aim of the phenomenological approach was to understand the meaning and essence of the participants' subjective experiences, as they lived through the course and various episodes of the pandemic. As a starting point, phenomenological interviews were conducted using general qualitative interviewing method, which was semi-structured in nature (25). Following Ricoeur (26), a phenomenological researcher is free to use structure in the interviews that enables a thorough investigation. The semi-structured approach was also advocated on grounds for maintaining methodological consistency and trustworthiness (25) especially in a study whereby three interviewers of varying research experience are involved. Due to constraints of the pandemic and for practical considerations, validation of the findings were not sought from the research participants as typically would be expected of the phenomenological method (27). Interviews were conducted in Singapore from October 29, 2020, to February 4, 2021. During this period of data collection, Singapore had no more active COVID-19 clusters of outbreaks. The country entered Phase 3 of re-opening on 28 December, 2020 whereby several restrictions were eased, such as increasing the maximum number of people allowed for social gatherings from five to eight, increasing the capacity limits of premises, and allowing migrant workers to access the community more often (28). This study adhered to the COREQ reporting guidelines.

### 2.2. Participants

Convenience sampling was used to recruit participants, and this was done through word-of-mouth, emails, and advertisements. Additionally, snowball sampling was used to complement recruitment. For instance, the study team mobilised their network in the field of geriatrics to recruit older adults. Potential participants were subjected to screening *via* a phone call before being recruited into the study. Inclusion criteria included English-speaking and able to provide informed consent. Individuals who were not residing in Singapore during the pandemic period and were younger than 18 years old were excluded.

### 2.3. Procedure

All interviews were conducted online using a teleconference platform Zoom due to COVID-19 restrictions and were guided by a topic guide informed by the HSDF. Three researchers consisting of one male research fellow (CC), one female research officer (MK), and one male research officer (BT) conducted hour-long semi-structured interviews. The topic guide (see Appendix A) provided a list of key questions that the interviewers had to follow through, thereby ensuring some degree of consistency across the three interviewers. At the same time, the interviews, being semi-structured in nature, allowed interviewers to follow-up on questions that were deemed important based on the replies of the respondents. Given that adhering to a topic guide may possibly limit the time participants have to adequately express their opinions, the team engaged the participants with two to three follow-up questions in instances where they assessed that the participants had more to share about a particular point. Sub-questions within the topic guide were explored optionally depending on the pace of the interviews. All had educational qualifications in psychology while CC and MK had prior experience in qualitative research. Given the evolving nature of the pandemic, the researchers kept up to date on the local developments of the pandemic by immersing themselves on news updates and actively discussing with each other on issues that arose.

Study materials consisting of an information sheet and topic guide were sent to enrolled participants prior to each interview session *via* email. Study goals and procedures were explained to each participant, and verbal consent was obtained at the beginning of each interview. Each interview consisted of at least two researchers, one to facilitate the interview and the other to take notes. Investigators met after each interview to reflect on the interviews and discuss their findings based on notes taken. Interviews were audio recorded and transcribed verbatim by researchers from a partner institute. To ensure confidentiality, audio recordings were destroyed after checking transcripts for accuracy and transcripts were de-identified. Transcripts were not reviewed by participants and no repeat interviews were conducted. Grocery vouchers worth SGD$25 (USD$19) were offered as an incentive for participation.

### 2.4. Data analysis

Thematic analysis (29) was conducted using QDA Miner Lite to organise the data and identify common themes and sub-themes about fear, stigma, and misinformation. Some preliminary themes aligned with the topic guide were developed based on the HSDF while new themes were generated during the coding process. The domains of HSDF proposed by Stang et al. (16) provided a ‘common ground' for researchers to understand health-related stigma and these included (i) personal experiences of stigma, (ii) perception of stigma in society independent of personal experiences, (iii) personal beliefs about what drove stigma and misinformation (iv) factors that facilitate the reduction of stigma and (v) perception of stigmatising behaviours and discriminatory attitudes/behaviours. Two of the three researchers (MK and BT) coded the first three transcripts independently to extract common themes before meeting to refine the codes and cheque for consistency. Any conflicts on a code's content were discussed and refined until a common understanding of the code was achieved. The researchers then came to an agreement on a refined list of codes and continued with coding the rest of the transcripts. Due to a member of the research team leaving the study (MK), BT coded the transcripts with the refined codebook while CC reviewed the coded transcripts. Both researchers then met regularly over 4 months to resolve any conflicting opinions and to discuss themes until no new themes were generated.

### 2.5. Ethical review

Institutional Review Board (IRB) approval was obtained from the National Healthcare Group-Domain Specific Review Board (reference number 2020/00582), based in Singapore. All participants gave verbal consent prior to the start of the interviews and anonymity and confidentiality were maintained according to the IRB-approved study protocol.

## 3. Results

Thirty-one participants were recruited for the study through non-probability sampling. However as one participant did not choose to proceed with the interview after being successfully recruited and another had family members engaging with the responses during the interview, this resulted in a final sample of 29 participants. Participants were aged 23–80 years old and on average 56.45 years old (*SD* = 16.8). Participant demographics are available in Table 1.

**Table 1:** Participant demographics (*n* = 29).

**Characteristics**	***n* (%)**
**Gender**	
Male	13 (44.8)
Female	16 (55.2)
**Age (in years)**	
21–30	4
31–40	2
41–50	3
51–60	4
61–70	10
71–80	6
**Ethnicity**	
Chinese	21 (72.4)
Malay	2 (6.9)
Indian	5 (17.2)
Others	1 (3.4)
**Employment status**	
Full-time	11 (37.9)
Part-time	5 (17.2)
Retired	11 (37.9)
Unemployed	2 (6.9)
**Highest level of education**	
Master's/doctorate or equivalent	5 (17.2)
Postgrad diploma/certificate	1 (3.4)
Bachelor's or equivalent	10 (34.5)
Professional qualifications	3 (10.3)
Post-secondary	2 (6.9)
Polytechnic	2 (6.9)
Secondary	5 (17.2)
Primary	1 (3.4)

Six major themes were generated to explore the effects of the COVID-19 pandemic on misinformation, fear, and stigmatisation: (i) perception of stigma amongst respondents, (ii) experiences of stigma amongst respondents, (iii) views on what drove stigma and misinformation, (iv) facilitators in preventing and reducing stigma and misinformation, and (v) ageist attitudes towards older adults (see Table 2).

**Table 2:** Major themes, sub-themes, and participant quotes.

**Major theme**	**Sub-theme**	**Participant quotes**
Perception of stigma amongst respondents	-	“I don't think it's (stigma) widespread simply because Singapore is majority Chinese right by race. I think of it as one race that globally that would be kind of stigmatised. It would be the Chinese for sure, but because we are most of us well, most meaning like I don't know what Singapore is, 60, 70% of the country is Chinese ethnically. I think that that type of racism or stigmatisation is a lot less” -PB05 “I've read articles about people not being served in hostels because they're wearing the nurse's uniform or the ambulance driver, stuff like that, and I mean, it's understandable, but like, I feel like that's a bit too much. The way people act is a bit too much. It's to possibly, yeah, like, I think that's more of like a fear of COVID-19 that's just going out of control.” -PB11 “I don't think we, like, keep away from Bangladeshi man or Indian man but I do hear of friends who say that when they see these people, they will move away in the MRT you know. You come up from the MRT and then they will go to another door or something like that.” -PB29
Experiences of stigma amongst respondents	-	“I mean, I guess if it (being stigmatised) happens it happens, but there's no real, like, fear I guess of it.” -PB11 “…I truly believe that if I have travelled, for example, if I travel to a country where they may think that I'm from mainland China, you know because I've seen those on news, right. I think so but because, you know, and I'm in Singapore. So I don't get that (stigmatised).” -PB70
Views on what drove stigma and misinformation	-	“For example, I have a friend who was working in a hospital, but she's nowhere near any COVID-19 patients or anything like that, and just because she's wearing the hospital uniform, pretty much the only place she can eat without getting stared at is within the hospital food courts and stuff.” -PB11 “I personally think that no one is to blame because the no one wanted the spread of COVID-19 to happen, but of course, sensationalised news will say China tourists they probably caused this whole thing to happen.” -PB13
Facilitators in preventing and reducing stigma and misinformation	Trust in government and local news sources	“I guess if the sources are government or medically backed up by facts from authorities that you can trust, and they know what they're talking about, then it's more trustable. Then I will see that several sources exist and then I'll trust the several sources” -PB30
	Quality of information and timely updates	“My view is, of course, the whole island-wide, they (the government) do announce through media, radio and TV and all that, and they update you actually very, very frequently. And they give advice and guidance as how one should protect oneself and to prevent the spread. I think that is very, very important.” -PB34
	Well-educated and informed public	“There is too much information and there are also a number of sensationalised information, be it Singapore or overseas, so I think we will have to be discerning to as to reading such things, as to, whether or not they are factual or distorted information.” -PB13
Ageist attitudes towards older adults	-	“…older people, the seniors that you can see in sort of the hawker centres, the seniors between the age of 70 to 80, they just couldn't be bothered. They don't care. They wear their mask under the chin, when I approached them, I say, why don't you pull up (the mask)? They say, I'm already so old, anytime can die. The way they answer you, they do not realise that they can infect other people, they do not know the consequence of infecting their own family, so they just don't care, the attitude is very complacent.” -PB35

### 3.1. Perception of stigma amongst respondents

Although participants perceived that the virus originated from Wuhan, China, they did not report China visitors (e.g. tourists or students) as being criticised in Singapore. One participant attributed this to the fact that majority of Singaporeans are of Chinese ethnicity. Another participant reported that locals were wary of the virus *per se* rather than the humans (China visitors) that may harbour the virus. One participant was mindful that unlike overseas countries in the West, the label “China Virus” did not exist in the local context and was a political construct. Many participants were aware that the “Chinese origin” narrative originated from the United States.

In Singapore, blue-collar workers typically in the construction field come from overseas and reside in designated large-scale dormitories. The cramped living conditions meant that large infection clusters quickly formed in these dormitories during the initial stage of the pandemic (30). At the time of the outbreak, there were 323,000 dormitory dwellers in Singapore (31). Our interviews showed that many participants were aware that COVID-19 impacted dormitory workers significantly. Beyond facing quarantine measures and movement restrictions, they were perceived to be stigmatised, and shunned by the public. A participant mentioned:

“I think the foreign workers are stigmatised especially when cases in dormitories are very high, I think Bangladeshi workers, they are pretty much stigmatised… I also do receive complaints from Singaporeans in saying that they have concerns about Bangladeshi cleaners and have they done swab test.” (PB13).

This arose from their perception that a large number of dormitory workers had been infected. A participant felt that dormitory workers were potentially shielded from discriminatory behaviour arising from stigmatisation only because they were kept quarantined in their dormitories. Hence, the situation may have been otherwise if they were not quarantined.

Healthcare workers were initially perceived to be at higher risk of being exposed to the virus and many were therefore shunned by the public. In particular, those wearing hospital uniforms were deemed to be stigmatised. A participant mentioned:

“Earlier, near the start there were some local news about nurses being asked to...leave the public transport or the bus or the train or there were videos of neighbours, you know, spraying alcohol or disinfectant at people who were... nurses who were coming back home.” (PB50).

Most participants however did not think this was persistent as it occurred mostly at the start of the pandemic. Although largely confined to healthcare workers, one participant opined that other frontline workers such as teachers and prison staff could have also been targeted given the nature of their work.

### 3.2. Experiences of stigma amongst respondents

Overall, the majority of the participants did not report experiencing stigma or being in a situation whereby they personally witnessed someone else being stigmatised. Most participants also did not experience fear in relation to stigma or discrimination and some mentioned that even if they became infected, they expected that existing family support would reduce the fear of stigmatisation. Strong family support therefore appears to be an important protective factor for many of the participants. However, some participants were wary of situations where they could be stigmatised. These included situations such as being infected with COVID-19, having to wear uniforms similar to frontline workers and being mistaken as someone from China while travelling outside Singapore. One participant mentioned that he would fear being Chinese in “Vancouver, United States or United Kingdom” (PB05). A few participants, however, mentioned that they felt avoided or discriminated as illustrated in the following examples: a participant was visibly ill in public, and she felt being “shunned”; another participant mentioned being “called out” by family members due to her job as a frontline worker: “…I've been told please do not carry the virus back home and infect the rest of the household.” (PB19).

It was possible that some participants dealt with their fear by avoiding places that had confirmed cases of COVID-19. For instance, some felt that they avoided places out of prudence such as specific shopping malls frequented by dormitory workers, or churches in Singapore with confirmed cases. This was illustrated by a participant:

“I, was scared to go. You know, for a few days we heard that [shopping mall A] got, [a COVID cluster] you know…we don't go [there] very often unless we really need to go there to get something. We faster go, no distance. We save distance faster get and come. We don't go anyhow, [go shopping mall A] or where. They said even [shopping mall B] have… [and] I stopped going [shopping mall B].” (PB64).

### 3.3. Views on what drove stigma and misinformation

One of the main drivers behind perceived stigma on dormitory workers and frontline workers involved the fear of infection. However, participants clarified that this was not due to some inherent characteristics of the groups but out of fear of the infection itself. It was considered prudent to “protect” oneself through avoidance behaviour, which was not regarded as discriminatory. As one participant remarked: “I would avoid going to places where there is a congregation of dormitory workers. But I wouldn't discriminate against them. I would avoid them, but not discriminate against them. The avoiding and discrimination are two different things.” (PB38). There is also an element of risk calculation driving the fear especially for healthcare workers who may have to “subject themselves to COVID-19 (in the care of patients)” (PB17) and that “health care personnel are the high-risk carrier…” (PB17).

Regarding concerns over overseas Chinese at the start of the pandemic, participants' responses suggest that there were initial concerns that Chinese tourists could have been vectors bringing in the virus. However, many also surmised that news about the origin of the virus could have caused this perception, which could have been subsequently amplified by unsubstantiated views promulgated by various information sources. As one participant mentioned:

“Initially there was all of these conspiracy theories that maybe the US who did it to China, maybe is China, who was researching stuff and they ran out of the laboratory, that kind of stuff. I don't know what to believe anymore…” (PB05).

Another participant said “I did read about accusations flying here and there. Some say Chinese, some say the American soldier, some say animal. Yeah. But, you know, there is no proof of anything...” (PB43). Interestingly, some participants felt that the Chinese were unfairly blamed, and this could possibly have portrayed Chinese excessively negatively. One participant mentioned: “…doesn't really help that the U.S. president has certain opinions about certain groups, especially the China (Chinese) people, so the people who buy into that, that will fuel their misinformation.” (PB22). Another participant, as with others, disagreed with the “Chinese/Asian origin narrative” and shared that the association of COVID-19 with one's ethnicity was a misinformed perception that could have been propagated by news or media: “…if you read the news and certain social media outlet…misconstrued that the virus very much has Asian origins” (PB19).

Beyond the perception that older people face significant risk of developing severe illness if they were infected with COVID-19 that could have explained the vulnerability narrative, there were views shared by participants that suggested that older adults were also more susceptible to misinformation. For instance, one participant, PB30, mentioned that older adults tend to spread misinformation on folk remedies to cure COVID-19 (e.g. basking in sunlight, drinking hot water). One participant perceived that older adults “take everything at face value” (PB13).

### 3.4. Facilitators in preventing and reducing stigma and misinformation

#### 3.4.1. Trust in government and local news sources

Most of the participants mentioned trusting the Singapore government and local news sources for information. Regarding trust in the government, there was a perception that information communicated to the public tended to be factual and reliable. One participant remarked:

“In my country, we have to trust the government or the government agency, because I think this is the most reliable source of information, because there is no guarantee that you'll send me whatever on social media that has been proven correct”. (PB39).

There were also laws protecting citizens from falsehood as highlighted by a participant: I have seen how my own country managed information, right? So ok, in Singapore we also have got laws very strictly barring against, you know, the spread of falsehoods.” (PB70). On local new sources, most participants mentioned trusting the information coming from local news platforms such as Channel News Asia and Straits Times. A participant felt that there is no politicising of issues: “Anything that's from Singapore, I'm inclined to agree. Only some, like if I watch Fox News and the CNN, that sort of news, I'm not too sure, because they seem to be ‘pro' certain things”. (PB70).

#### 3.4.2. Quality of information and timely updates

The manner through which high-quality information was readily disseminated to the public was another possible factor that could have reduced misinformation. Many were familiar with the type and manner of updates that they received. For instance: “When you have daily updates at the time, you know that you will get update on numbers, on developments at that time. So that removes the vacuum of information in which misinformation can spread” (PB 05). Others, e.g., PB13 and PB16, mentioned about the benefits of a “daily/regular press conference and press releases” from the government.

#### 3.4.3. Well-educated and informed public

Finally, participants perceived that a well-educated and informed public could have also helped in discerning the information they received, hence reducing stigma and related misinformation. Participants mentioned checking and verifying information that they receive. One participant, PB38 would first establish credibility of who was making the statement before agreeing with it whereas another, PB40, would “fact-check” by using search engines such as “Google”. Participants tended to be more cautious if the information they come across was overly negative or if the source was from social media.

### 3.5. Ageist attitudes towards older adults

Expressions of concern were common when participants were questioned about the impact of the pandemic on older adults. There was a general sense that older adults were a homogeneous vulnerable group and were more in need of help than younger people. A participant mentioned: “Number one, they're more susceptible. Number two, they probably are more fearful. Number three, they're probably more susceptible to misinformation as well. So emotionally, economically… I mean, on all fronts, they are the ones who are losing out here.” (PB05).

The responses also included perceptions of how older adults were dealing with the pandemic. There was a sense that older adults were less able to cope with changes in their lives: “So there was a lot of unacceptance... and they couldn't accept this at all. They couldn't accept this, all this rules. The old people, …it was very sudden for them, and they couldn't accept it.” (PB21). There was also perception of helplessness and the inability of older adults to competently care for themselves. One participant mentioned that the older adults were misinformed: “…the seniors are not getting the exact information from the media and they communicate with their group and that must be a lot of misunderstanding, a misinterpretation of the policy.” (PB73). One participant, PB19, a volunteer at a care centre, mentioned that seniors complained that their movements were restricted by their family out of concern for their vulnerability.

Overall, our study showed that younger participants and to some extent, older ones too, tended to subscribe to the vulnerability narrative of older adults. However not all older adults felt this way. Some were unhappy about ageist attitudes that surfaced because of the pandemic:

“Well I find it like come on, doesn't mean that I am of this age, I am vulnerable, you know. I don't think they should brand us (older adults) that way, which is very, very bad, very hurting. My children also follow along because the news is saying that, you know so they keep cautioning me, don't go out, don't go out, don't go.” (PB21).

## 4. Discussion

Adapting elements of the Health Stigma and Discrimination Framework, this study delved into understanding the stigmatisation process that occurred in Singapore during the early phase of the pandemic and not only examined manifestations of stigma in the form of perception, experiences and practises, but also identified the drivers and facilitators behind how stigma is applied to certain groups according to their race or occupation.

Findings from this sample suggest that some groups were perceived to have been stigmatised by the public during the start of the pandemic. These included healthcare workers and dormitory workers with the former being widely reported in existing literature (32–35). As with earlier studies during the SARS outbreak, drivers of stigma against healthcare workers identified in this study was similar as they were shunned and ostracised for fear that they were potential carriers of the virus (36, 37). Initiatives in Singapore to recognise the efforts of healthcare workers as well as a narrative on their sacrifices and contributions as the pandemic progressed, could have had a positive effect in reducing stigmatisation (35).

Stigmatisation of dormitory workers occurred as they formed the vast majority of cases earlier in the pandemic where the virus spread quickly due to their communal living arrangements (38). The main driver behind the pattern of stigmatisation was similar to healthcare workers insofar as this group was perceived to be potential carriers of the virus. There was a general sense that avoidance behaviour was the prudent thing to do, similar to what was observed in the United States and Canada (33). Interestingly, the interviews did not reveal any deep-rooted anger or hatred towards dormitory workers for the large increase in infection numbers. They were also not perceived as “scapegoats” that were to be blamed given their status in society (39). This was in a way surprising given that other studies have shown that ostracism or other forms of discriminatory practises would be expected in a pandemic (40–42).

Prior to the pandemic, attitudes towards dormitory workers were not always positive as revealed in a survey conducted by the International Labour Organisation (ILO) (43). For instance, in 2008, residents of an estate had signed a petition against a foreign workers dormitory situated in their neighbourhood (44). Moreover, these workers are often viewed as a forgotten segment of society whereby their poor living conditions were not a focus of attention until the pandemic hit (44). There was therefore the possibility that some participants in our sample could have offered socially desirable comments. Alternatively, since this study employed the use of convenience and snowball sampling, it was also possible that participants of certain traits and viewpoints may have self-selected themselves to participate in the study. These views therefore reflected the thinking from segments of society that did not hold strong views against dormitory workers.

With regard to relatively absent anti-Chinese national sentiments, a possible reason on why participants in our sample were mindful of the “Chinese origin” narrative of the virus could be in part due to local political leaders actively taking the stand against anti-Chinese sentiments that initially surfaced, largely framing this as a medical issue, staying clear of terms such as “Wuhan virus” that could feed such sentiments (45). To surmise, our findings concurred with earlier studies related to pandemics, where the fear of contracting the virus led to the stigmatising of groups known to be largely infected or suspected to be so due to close contact with the latter (5, 39).

Despite the perceived existence of stigma against groups known to be at high risk of infection, our participants did not reveal much experienced stigma (personally experiencing incidents or knowing of cases from personal networks). There were however views that highlighted how people would fear being mistaken as uniformed frontline workers or as someone coming from China. For the latter group, this fear was driven by the global perception that people of Chinese descent have overwhelmingly been the target of discrimination largely because of the negative portrayal of the Chinese, which was promulgated by overseas news portals, social media, and prominent public figures in the United States. The perception coming from our sample that prominent figures in the United States might have exacerbated the stigmatisation of the Chinese has been surfaced in other studies (14, 46).

Participants mentioned that government and local news outlets in Singapore were trusted sources for accurate information related to the pandemic. This could possibly explain why they were mindful of misinformation surrounding Chinese individuals and other associated stigmatising practises. Indeed, studies have found that lower trust in the government to be a predictor of higher susceptibility to misinformation (7, 47). Views on trust towards the Singapore government in communicating information about COVID-19 corroborated with empirical data provided by a separate study (48). In this pandemic, beyond regular communications and prompt correction of misinformation by the government, fake news law passed have been reported by the home affairs minister in helping to substantially reduce the circulation of misinformation (49). Such proactive approaches in keeping the public informed could also be effective at reducing belief in misinformation through a process known as ‘cognitive inoculation' (50). Given emerging evidence suggesting that misinformation can influence people's behaviour negatively during the pandemic, such as lowered willingness to adopt public health guidance measures, more than ever, public institutions involved in fighting the pandemic must continue to gain the trust of the public as reliable sources of information, by providing regular and timely updates so as to limit the spread of misinformation.

The role played by traditional mainstream media is however not always clear. Elsewhere in the United States, it has been found that those who disproportionately consumed right-leaning media were more likely to endorse COVID-19 misinformation (51). Other research showed a positive association between exposure to traditional media and lower misinformation beliefs (52). More recently, exposure to traditional media was found to have a positive association with vaccine acceptance (53). As participants viewed the information coming from news outlets in Singapore to be direct, factual, and non-sensational, and therefore had a level of trust in it, this may have contributed to participants' ability to distinguish between misinformation (e.g., origin of virus, folk remedies) and factual information. In line with recommendations from other research (52), traditional media should continue to adhere to disseminating fact-based information linked to high quality sources such as governmental, healthcare or academic data and reports.

Some studies suggested that education level did not play a role in predicting whether someone will believe in misinformation (53, 54). The evidence on the role of education was not clear as it was not the focus of our study although we uncovered that strategies used by our highly educated sample such as active fact-checking and verification of sources were likely important in combating misinformation. Findings from this study point to the benefits of multi-modal means of messaging during the pandemic by official governmental sources. Future research could examine the role of community leaders and religious leaders in information dissemination efforts as they have been suggested by some of those who were more religiously inclined in our study as possibly playing a role in complementing governmental sources.

Participants' responses also suggested that some may hold certain ageist assumptions of the older population. These attitudes appeared to have been benevolent and paternalistic in nature, stemming from concern towards older adults to care for and protect them (55, 56). Public health messaging therefore needs to be designed in a way that does not further exacerbate benevolent ageism in the community, such as by framing messaging that does not homogenise older adults that could fit paternalistic age stereotypes (57). As the messaging has already been done and protracted, policymakers should focus future communication on dialling down the effects of COVID-19 public health messaging targeting older adults, such as the widely adopted “vulnerability” narrative (56).

Lastly, many participants reported preferring a multi-modal approach with a focus on video and text-based messages (e.g. through platforms such as Telegram and Whatsapp) although some mentioned the latter could take up too much time and may be unsuitable for some segments of the population such as older adults or those with lower health literacy. Infographics were also mentioned as useful ways of conveying important information. Majority of participants prefer receiving information through official sources such as press briefings and government linked websites. Information should also be disseminated through all mediums including print, broadcast, and news media. Regarding messengers, other than through local authorities and news channels, experts such as doctors and other reputable figures have been suggested as figures who could facilitate information dissemination.

## 5. Limitations

This study was not without limitations. Overall, our participants were well-educated, and many were discerning of news and information they receive. Over-representation of particular groups as in our study is not uncommon given the use of non-probability sampling and views on misinformation stigma, and fear during the pandemic may therefore differ should there by greater heterogeneity in education level. Interviews were also all conducted in English *via* Zoom, which meant that participants in our sample also possessed a certain level of digital literacy. This was also the case for the older adults in our sample where digital literacy is typically instead much lower as shown in a recent local study (58). Many of the older adults in our sample were familiar with social media and actively subscribed to various official news platforms in the digital sphere such as Twitter, Facebook, WhatsApp, and Telegram. Therefore, views from older adults in this sample could differ from those in the population with lower digital literacy.

Given the sensitive nature of the topic, participants could potentially have withheld or altered their opinions on stigma due to social desirability effect especially when probed about their views relating to foreign workers. Moreover, since foreign dormitory workers were not interviewed in this study, views from participants in this study about the lack of ostracism or discriminatory practises against dormitory workers could not be corroborated. Indeed, whilst the study was able to examine the key domains under the HSDF, namely the manifestations of stigma and the various driver and facilitators, not reaching out to the affected population meant that the impact of stigma on access to justice, uptake of testing, adherence to treatment, resilience and advocacy (16) could be further explored. Future research using the HSDF should therefore pay closer attention to understanding such outcomes beyond the focus on the other domains. Finally, these participants were interviewed at a time when COVID-19 situation was generally under control. Given the evolving nature of the pandemic, attitudes and opinions could differ if the interviews were conducted at an earlier stage of the pandemic whereby there was more uncertainty about the covid-19 cases involving dormitory workers.

## 6. Conclusion

This study explored the perceptions and experience of the laypersons on stigma and identified stigma drivers and facilitators during the COVID-19 pandemic. Perceived stigma existed largely towards dormitory workers and healthcare workers. Personal experiences of stigma were not widespread and while majority of participants reported being unafraid of stigmatisation, some were cautious of situations where they could be stigmatised. Key drivers of stigma and misinformation were identified, such as fear of infection and overseas information sources. Trust in local sources for information, fact-checking, and the manner of information dissemination were suggested to facilitate the prevention or reduction of stigma and misinformation. An important next step would be to utilise the findings to guide development of strategies and tools, such as in public health messaging, to combat the spread of stigma and misinformation in future pandemics.

## Data availability statement

The datasets presented in this article are not readily available because as stipulated by the ethics board, sharing raw data beyond the study team members is not permitted. However, further inquiries can be directed to the corresponding author for clarifications. Requests to access the datasets should be directed to yu.chou.chuen@geri.com.sg.

## Ethics statement

The studies involving human participants were reviewed and approved by the National Healthcare Group Domain Specific Review Board (Reference number 2020/00582). The patients/participants provided their written informed consent to participate in this study.

## Author contributions

JL and CY are the co-investigators of the study and made significant contribution to study design, investigation, and reviewing of this manuscript. CY and BT made significant contribution to study design, investigation, analysis, study administration and writing of this manuscript. CF, MM, and SS made significant contribution to study design and reviewing this manuscript. CY made major contribution to writing this manuscript. All authors have read and approved the manuscript.
