# Supplementary material for: A qualitative study on health stigma and discrimination in the first year of the COVID-19 pandemic: Lessons learnt from a public health perspective
Source: Front Public Health. 2023 Mar 1;11:1143640. doi: 10.3389/fpubh.2023.1143640 (PMC10014793; doi:10.3389/fpubh.2023.1143640)
Supplement: Supplementary file 1 [file Data_Sheet_1.pdf]

Appendix A

Section 1: General Questions

1) What is your current knowledge about the novel coronavirus or COVID-19 [*use the term participant uses*]?

1.1. How did you first learn about COVID-19?
1.2. Do you feel as if you have enough information on COVID-19?
1.3. Is there any information about COVID-19 that is confusing? If so, can you describe?

2) How have you been obtaining your information regarding COVID-19?

2.1. What type of information were you most interested in learning about COVID-19?
2.2. What sources (e.g., online media, news channels, family and friends, etc.) have you been using to get your information?
2.3. Are these the sources you typically use to obtain news/information?
2.4. What challenges have you experienced in terms of seeking information about COVID? (e.g. too much information)
2.5. What has been helpful for you in terms of seeking information about COVID? (e.g. transparency of information from government, gov.sg group chats)

3) Could you describe your perceptions of COVID-19? Do you think the public/other people have the same reaction? (Optional if not enough time)

4) How has COVID-19 impacted you personally?

4.1. How has it impacted your day-to-day life (e.g., job, health, family)?

5) What is your greatest fear as related to COVID-19?

5.1. What are additional fears or concerns that you have?

Section 2: Drivers

Please think about your concerns and feelings since the beginning of the novel coronavirus or COVID-19 pandemic when you answer the next few questions.

1) What are your thoughts on the economic impact COVID-19 will have on you or on others?

1.1. What are the economic impacts on older adults (65 and above) vs younger adults?

2) Who would you feel comfortable reaching out to for help/support because of COVID-19 impacts?

3) Are there aspects of your identity (e.g., race, age, gender, sexual orientation) that has impacted your willingness to seek help if you thought that you were ill with COVID-19?

Section 3: Facilitators

Please think about what has influenced your views since the beginning of the COVID-19 pandemic when you answer the next few questions.

1) You have been sharing your views on COVID-19 outbreak, can you please describe any factors that have influenced your views during the COVID-19 outbreak?

2) What do you think about the guidance being put out (e.g., washing your hands, social distancing, etc.)?

3) Do you think certain groups in the public may experience health consequences by COVID-19 more negatively than others?

3.1. If yes, please describe
3.2. What would be the health impacts of older adults (65 and above) vs younger adults?

4) What are your thoughts of the health policies that have been put in place to combat the spread of COVID-19?

Section 4: Stigma Marking

In this section, we're looking to discuss how stigma has impacted different groups since the COVID-19 outbreak.

Example of stigma: Asians being attacked/harassed overseas because they were thought to be Chinese

1) Do you think there is someone responsible for COVID-19? If yes, who do you think gets the most blame for COVID-19?

1.1. What have you seen, heard, or read that makes you feel this way?

2) Do you think fear and misinformation around COVID-19 has been targeted towards certain groups?

2.1. If yes, what aspects of someone's identity, such as race, age, gender or sexual orientation, have been stigmatized because of COVID-19?
2.2. What are your thoughts on the accuracy of that information?
2.3. What are your feelings towards how certain groups are targeted?

3) Do you agree with the media/news (e.g., social media, news articles, TV broadcasts) on the information being shared on COVID-19? Why or why not?

Section 5: Stigma Experiences

Please think about any experiences on stigma since the beginning of the COVID-19 pandemic when you answer the next few questions.

As a result of the recent COVID outbreak…

1) Have you experienced any discrimination because of COVID-19?

   1.1. If you're comfortable, could you share what that experience was like?

2) How do you think people who are stigmatized are doing given the spread of COVID-19?

   2.1. How has this impacted their personal/social life?
   2.2. How has this impacted their professional life?
   2.3. What kinds of changes do you think they have had to make because of this stigmatization?

3) Do you fear experiencing stigma because of COVID-19? [If answer no to question 1]
